# The complete mitochondrial genome of the *Acropora pruinosa*

**DOI:** 10.1080/23802359.2017.1375882

**Published:** 2017-09-11

**Authors:** Peng Tian, Wentao Niu

**Affiliations:** Laboratory of Marine Biology and Ecology, Third Institute of Oceanography, State Oceanic Administration, Xiamen, China

**Keywords:** Coral, mitogenome, phylogeny

## Abstract

In this study, the complete mitogenome sequence of stony coral, *Acropora pruinosa* (Scleractinia), has been decoded for the first time by next generation sequencing and genome assembly. The assembled mitogenome, consisting of 18,480 bp, has unique 13 protein-coding genes (PCGs), three transfer RNAs, and two ribosomal RNAs genes. The complete mitogenome of *Acropora pruinosa* showing 99% identities to *Acropora nasuta*. The complete mitogenome provides essential and important DNA molecular data for further phylogenetic and evolutionary analyses for coral phylogeny.

The Acroporidae is one of the most important families of reef corals. *Acropora pruinosa*, one of the members of Acroporidae family, is widely distributed throughout the Indo-Pacific. It is common in most reef environments, especially in shoal water. The first establishment of *A. pruinosa* mitogenome is important for further evolutionary and phylogenetic analyses for stony coral (Chen et al. 2008).

Samples (voucher no. DYW9) of *Acropora pruinosa* were collected from Daya Bay in Guangdong, China. We used next generation sequencing to perform low-coverage whole genome sequencing according to the protocol (Niu et al. 2016). Initially, the raw next generation sequencing reads generated from HiSeq 2000 (Illumina, San Diego, CA). About 0.09% raw reads (48,043 out of 339,941,168) were *de novo* assembly by using commercial software (Geneious V9, Auckland, New Zealand) to produce a single, circular form of complete mitogenome with about an average 582× coverage.

The complete mitogenome of *Acropora pruinosa* was 18,480 bp in size (GenBank KY094483) and its overall base composition is 25.0% for A, 13.77% for C, 24.33% for G, and 36.9% for T, and has GC content of 38.1%, showing 99% identities to *Acropora nasuta* (GenBank KF448536.1). The protein coding, rRNA, and tRNA genes of *Acropora pruinosa* mitogenome were predicted by using DOGMA (Wyman et al. 2004), ARWEN (Laslett and Canback 2008), MITOS (Bernt et al. 2013) tools and manually inspected. The complete mitogenome of *Acropora pruinosa* includes unique 13 protein-coding genes (PCGs), three transfer RNA genes (tRNA-Met, tRNA-Aln, and tRNA-Trp), and two ribosomal RNA genes. All PCGs, tRNA, and rRNA genes were encoded on H-strand. The PCG of NAD5 has a 12,071 bp intron insertion. It is important to note that two PCGs started with ATG codon (ATP6 and ATP8), one with ATT codon (ND5), one with GTG codon (ND4L), two with TTG codon (COX2 and Cyt b), one with ATA codon (ND6), and six with TTA codon (ND1, ND2, ND4, COX3, COX1, and ND3). Five of 13 PCGs are inferred to terminate with TAA (ND1, ND2, ND6, ND4L, and COX1), eight PCGs with TAG (Cyt b, ATP6, ND4, COX3, COX2, ND3, ND5, and ATP8). Among 13 PCGs, the longest one is ND5 gene (1686 bp), whereas the shortest is ATP8 gene (219 bp). There were 14 bp overlapping nucleotides between ND4L and tRNA-Aln, 10 bp overlapping nucleotides between ATP8 and COX1, and the number of non-coding nucleotides between different genes varied from 15 to 1227 bp.

To validate the phylogenetic position of *Acropora pruinosa*, we used MEGA6 software (Tamura et al. 2013) to construct a maximum likelihood tree (with 500 bootstrap replicates and Kimura 2-parameter model) containing complete mitogenomes of 10 species derived from Astrocoeiina of Scleractinia. *Turbinaria peltata* derived from Dendrophylliidae was used as outgroup for tree rooting. Result shows *Acropora pruinosa* is closely related to *Acropora nasuta* with high bootstrap value supported (Figure 1). In conclusion, the complete mitogenome of the *Acropora pruinosa* deduced in this study provides essential and important DNA molecular data for further phylogenetic and evolutionary analyses for stony coral phylogeny.

**Figure 1.**
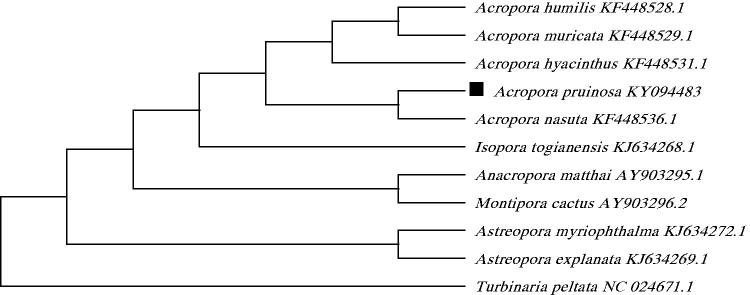
Molecular phylogeny of *Acropora pruinosa* and related species in Scleractinia based on complete mitogenome. The complete mitogenome is downloaded from GenBank and the phylogenic tree is constructed by maximum likelihood method with 500 bootstrap replicates. The gene's accession number for tree construction is listed behind the species name.
